# A Population-Based Cohort Study on Chronic Comorbidity Risk Factors for Adverse Dengue Outcomes

**DOI:** 10.4269/ajtmh.21-0716

**Published:** 2021-09-27

**Authors:** Chia-En Lien, Yiing-Jenq Chou, Yi-Jung Shen, Theodore Tsai, Nicole Huang

**Affiliations:** ^1^ Research Center for Epidemic Prevention, National Yang Ming Chiao Tung University, Taipei, Taiwan;; ^2^ Institute of Public Health, School of Medicine, National Yang Ming Chiao Tung University, Taipei, Taiwan;; ^3^ Office of the Deputy Superintendent, National Yang Ming Chiao Tung University Hospital, Yilan County, Taiwan;; ^4^ Institute of Hospital and Health Care Administration, National Yang Ming Chiao Tung University, Taipei, Taiwan;; ^5^ Takeda Vaccines, Cambridge, Massachusetts

## Abstract

The global burden of dengue is increasing against a background of rising global prevalence of chronic noncommunicable diseases (NCDs) and an epidemiological shift of dengue toward older age groups. The contribution of NCDs toward risk for adverse clinical and healthcare utilization outcomes was assessed in a national linked-database study. About 51,433 adult dengue cases between 2014 and 2015 were assessed for outpatient and inpatient claims data in Taiwan’s National Health Insurance Research Database for the 30 days after their dengue diagnosis. A multivariable logistic regression with generalized estimating equations was used to estimate the probability of adverse dengue outcomes in patients with NCDs compared with dengue patients without underlying diseases. Rheumatoid arthritis and related disease were associated with the highest risk of hospitalization after dengue diagnosis (odds ratio: 1.78; 95% CI: 1.37–2.30), followed by stroke, chronic kidney disease (CKD), liver cirrhosis, asthma, coronary artery disease, chronic obstructive pulmonary disease, diabetes, congestive heart failure, hypertension, and malignancy. Chronic kidney disease and diabetes were associated with higher risks of hospitalization, intensive care unit (ICU) use, and all-cause mortality. After adjusting for socioeconomic status and other variables, the number of coexisting chronic diseases was associated with increasing risk of adverse dengue outcomes. Specific NCDs were associated with longer hospitalizations, ICU admission, and higher healthcare costs. Quantifying the risks of adverse dengue outcomes and health expenditures among dengue patients with preexisting NCDs provides insights for improved clinical management and essential inputs for health economic analyses on the cost-benefit of risk-based routine or catch-up immunization programs.

## INTRODUCTION

Dengue is a mosquito-borne infectious disease caused by four dengue viruses (DENV) transmitted by *Aedes aegypti* or *Ae. albopictus*. It emerged as a global public health problem after World War II as a result of ecological disruption, global population growth, urbanization, and travel, and the geographical expansion of the respective vectors.^1^ The disease is transmitted in an endemic pattern in at least 128 countries, putting almost four billion people at risk annually.^2^ Amidst the global trend of declining communicable diseases,^3^ dengue stands out, having doubled in incidence every 10 years in recent decades. The most commonly cited estimate of dengue infection incidence is between 50 and 100 million annually.^4^ The growing burden has resulted in WHO’s listing dengue as one of the 10 threats to global public health in 2019.^5^

The disease’s clinical manifestations range from asymptomatic infection to life-threatening complications. There is no effective antiviral therapy and treatment remains supportive^6^ for the estimated 500,000 cases hospitalized for severe dengue annually. The case fatality rate (CFR) has declined with improvements in case management^7^ to, for example, a nationally reported CFR of 0.1% in Vietnam. Reducing dengue CFR, using a standardized CFR definition, is a WHO goal for the next decade.

The demographic transition of dengue, with an increasing proportion of adults among all, severe, and fatal cases of dengue^7^ poses a challenge for clinical management, as older adults are at high risk for complicated and fatal dengue outcomes.^8^^,^^9^ Analysis of mortality data from 52 countries showed an association between a country’s gross domestic product (GDP) per capita and the proportion of fatal dengue cases occurring in adults,^10^ suggesting that more countries may experience the same trend as their economies develop.

A systematic review of 47 studies showed elevated age-adjusted odds ratios (ORs) of severe dengue outcomes from 1.61 in people with hypertension, 2.14 in people with heart disease, 2.76 in people with diabetes, to 3.41 in overweight/obese people.^11^ Another systematic review of 243 studies analyzing dengue outbreaks in 1990–2015 reported that in addition to diabetes and hypertension, renal insufficiency and hypertension also were significant risk factors for severe dengue outcomes.^12^ Other studies suggest that underlying chronic kidney disease (CKD), chronic liver disease, or prior stroke were associated with higher risks for dengue hemorrhagic fever (DHF), dengue shock syndrome (DSS), and other severe or fatal dengue outcomes.^13^^,^^14^

The existing literature suggests a few research gaps. Because of cross-sectional designs, many failed to differentiate between chronic disease diagnoses occurring before, during, and after the dengue episodes. The lack of a clear temporal relationship between chronic conditions and dengue infection compromises their internal validity.^11^ Existing studies were mostly single-center or hospital-based, introducing the possibility of selection bias and only a few studies analyzed how more than one chronic comorbidity impacts dengue outcomes. Lastly, systematic literature reviews were limited by study heterogeneity in estimating effects on dengue severity.^15^

Elucidating the relationship between preexisting chronic conditions and severity of dengue outcomes can help clinicians identify vulnerable subpopulations for close monitoring and early intervention. From a public health perspective, such investigations can provide the basis for risk-based vaccine recommendations when a dengue vaccine is available.^16^ In this study, we use data linkages among the Taiwan National Health Insurance Research Database (NHIRD), the Notifiable Disease Dataset of Confirmed Cases (NDDCC) from Taiwan’s Infectious Disease Surveillance and Reporting System, and the vital statistics death certificate file to identify 51,433 laboratory-confirmed adult dengue cases in Taiwan. We then estimated the association between preexisting chronic conditions and dengue outcomes, including hospitalization, length of stay (LOS), medical expenditure, and mortality while adjusting for the presence of multiple noncommunicable diseases (NCDs). The study is the first, to our knowledge, to use a population-based dataset that assesses the impact of multiple preexisting chronic conditions on clinical outcomes and medical expenditures among adult dengue patients while controlling for socioeconomic status (SES).

## MATERIALS AND METHODS

### Data source and study sample.

**Figure 1.**
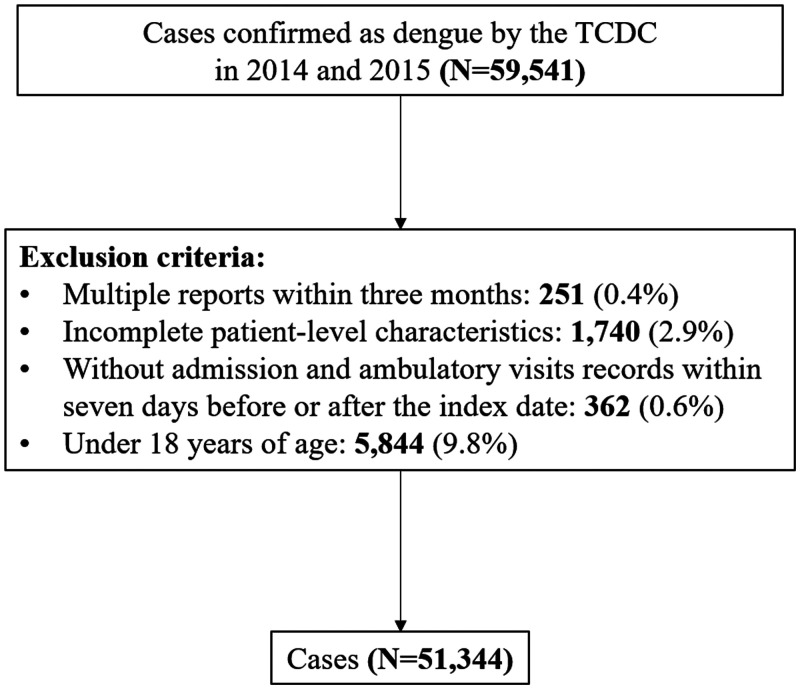
Flow chart of dengue sample selection. TCDC = Taiwan Centers for Disease Control.

The NDDCC is estimated to include up to 96% of all confirmed dengue cases.^22^ The NHIRD, covering 99.5% of the Taiwan population, was linked to provide information on patient characteristics, diagnosis of the chronic condition, and healthcare utilization. The validity and quality of the NHIRD have been assessed repeatedly.^17^^,^^18^ Index dates were defined from NDDCC records and death certificates provided date and causes of death. Our sample included adults aged above 18 years with confirmed dengue occurring between January 1, 2014, and December 31, 2015, during the largest dengue outbreak in Taiwan since 1998. Among 59,541 confirmed dengue cases in the study period, we excluded 251 (0.4%) having multiple reports within 3 months, 1,740 (2.9%) with incomplete patient-level characteristics, 362 (0.6%) without admission and ambulatory visit records within 7 days before or after the index date, and 5,844 (9.8%) who were under 18 years of age (Figure 1).

### Dependent variables.

Each confirmed dengue patient was followed for 30 days after the index date of dengue confirmation for multiple outcomes: hospitalization, intensive care unit (ICU) admission, and death. To better understand resource utilization associated with dengue infection, the length of dengue-related hospital stay (LOS), inpatient and total medical care expenditures within 30 days of dengue confirmation index date also were assessed. Dengue-related hospitalization was defined as inpatient admissions with any diagnosis of dengue of ICD-9-CM codes 061, 065.4, 066.3, and V73.5. Multiple admission records during the follow-up period were combined. Intensive care unit admissions during dengue hospitalization were identified by inpatient claims records. Thirty-day all-cause mortality comprised deaths occurring within 30 days of the index date. Length of stay and inpatient expenditure for each dengue admission were calculated. Total medical expenditure incurred by each dengue patient included inpatient, outpatient, emergency, and prescription drug expenditures within the same dengue episode.

### Noncommunicable diseases.

**Figure 2.**
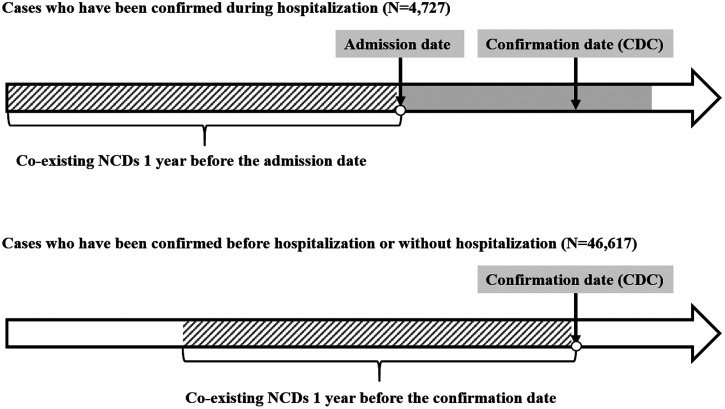
Definition of NCDs collection. CDC = Centers for Disease Control; NCDs = noncommunicable diseases.

Based on literature reviews and burden of disease by cause in East Asia, 13 NCDs were analyzed: malignancy, diabetes, coagulation and hemorrhagic disorders, hypertension, coronary artery disease, congestive heart failure (CHF), stroke, chronic obstructive pulmonary disease (COPD), asthma, rheumatoid arthritis (RA) and related disease, CKD, major depressive disorder, and liver cirrhosis (Supplemental Table 1 provides ICD-9-CM codes).^19^ Principal and secondary diagnoses identified underlying NCDs. Inpatient admission or outpatient clinic attendance three times or more 1 year before the index date for any of the above specific NCD defined a diagnosis with a particular NCD (Figure 2.). Stroke was defined by hospitalization for stroke within 1 year before the index date. Additionally, we determined the number of distinct NCDs per patient using the clinical classification system (CCS), developed by the Healthcare Cost and Utilization Project (HCUP).^20^ The CCS classified all ICD-9-CM diagnosis codes into 260 mutually exclusive, clinically homogeneous categories. We classified each patient into four categories based on their total number of chronic conditions (0, 1, 2, 3, or more).

### Patient characteristics.

The patients’ age, sex, and SES were included for adjustment and subgroup analyses. Patient age was stratified from 18 to 44, 45 to 64, 65 to 74, > 75 years. Socioeconomic status was defined by the primary insurance holder’s insurable wages and types. People with a well-defined monthly wage were classified into three categories: < $968, $968–$1,935, and ≥ $1,935. A small number without well-defined monthly wages were identified separately.

### Statistical analysis.

Simple descriptive statistics were computed to describe rates of dengue outcomes, the average LOS, and medical expenditure. Multiple logistic regressions were used to examine the association between multiple NCDs and dichotomous outcome variables (hospitalization, ICU admission, death). Multivariate linear regression models with log transformation of outcomes were applied to assess NCDs’ influences on LOS and medical expenditure. The outcomes of ICU use, length of hospital stay, and hospitalization expenditure were analyzed among patients who had been admitted for dengue. Patient characteristics and the index year were included as control variables in the models. Stratification analyses were also conducted to assess the effects of NCDs by age. We described all cases over 18 years of age but limited the analysis of NCD risk to patients >45 years of age because of their low prevalence in younger patients. Sensitivity analyses were also conducted for all-cause hospitalization and death using different modeling strategies such as two-part models. The main results remained robust. The analyses were adjusted for robust standard errors. All analyses were conducted using SAS 9.4 and STATA 15.

## RESULTS

Among 51,433 confirmed adult dengue patients in Taiwan between January 1, 2014, and December 31, 2015, 60.1% were 45 years and older. Over half of the patients (67.6%) had no coexisting NCDs (Table 1). The topmost prevalent NCDs among dengue patients were hypertension (23.1%), diabetes (11.6%), and coronary artery disease (4.8%). Among all and NCD patients, respectively, 30.9% and 44.7% patients were admitted for dengue within 30 days of the NDDCC index date (Table 2) 1.7% and 4.0% required ICU care and 373 (0.7%) and 326 (2.0%) died within 30 days after the diagnosis. The incidences of these adverse outcomes increased with patient age. The average LOS was 6.11 days. The average inpatient expenditure for hospitalized patients was $782.65. The average total medical expenditure for all dengue patients including many patients who only used outpatient care was $379.27. Older patients were found to need a longer hospital stay, and incurred higher inpatient and total expenditures.

**Table 1:** Demographic characteristics of patients with dengue in Taiwan, 2014–2015

	Total	Age (years)			
	(*N* = 51,344)	18–44 (*N* = 20,427, 39.8%)	45–64 (*N* = 19,587, 38.1%)	65–74 (*N* = 7,058, 13.7%)	≥ 75 (*N* = 4,272, 8.3%)
Sex					
Male	25,100 (48.9%)	10,830 (53.0%)	8,955 (45.7%)	3,271 (46.3%)	2,044 (47.8%)
Female	26,244 (51.1%)	9,597 (47.0%)	10,632 (54.3%)	3,787 (53.7%)	2,228 (52.2%)
SES, USD	–	–	–	–	–
< $968	19,514 (38.0%)	10,050 (49.2%)	6,594 (33.7%)	2,038 (28.9%)	832 (19.5%)
$968–$1,935	13,768 (26.8%)	4,874 (23.9%)	6,402 (32.7%)	1,717 (24.3%)	775 (18.1%)
≥ $1,935	3,666 (7.1%)	1,208 (5.9%)	1,560 (8.0%)	505 (7.2%)	393 (9.2%)
Fishers or farmers	4,864 (9.5%)	1,067 (5.2%)	1,941 (9.9%)	1,060 (15.0%)	796 (18.6%)
Others	9,532 (18.6%)	3,228 (15.8%)	3,090 (15.8%)	1,738 (24.6%)	1,476 (34.6%)
Comorbidities	–	–	–	–	–
Malignancy	2,072 (4.0%)	172 (0.8%)	912 (4.7%)	541 (7.7%)	447 (10.5%)
Diabetes	5,956 (11.6%)	245 (1.2%)	2,380 (12.2%)	2,003 (28.4%)	1,328 (31.1%)
Coagulation and hemorrhagic disorders	190 (0.4%)	41 (0.2%)	63 (0.3%)	79 (1.1%)	7 (0.2%)
Hypertension	11,864 (23.1%)	547 (2.7%)	4,938 (25.2%)	3,678 (52.1%)	2,701 (63.2%)
Coronary artery disease	2,484 (4.8%)	68 (0.3%)	852 (4.3%)	850 (12.0%)	714 (16.7%)
Congestive heart failure	443 (0.9%)	18 (0.1%)	110 (0.6%)	135 (1.9%)	180 (4.2%)
Stroke	329 (0.6%)	19 (0.1%)	103 (0.5%)	104 (1.5%)	103 (2.4%)
COPD	1,157 (2.3%)	96 (0.5%)	359 (1.8%)	330 (4.7%)	372 (8.7%)
Asthma	698 (1.4%)	110 (0.5%)	271 (1.4%)	168 (2.4%)	149 (3.5%)
Rheumatoid arthritis and related disease	253 (0.5%)	46 (0.2%)	111 (0.6%)	68 (1.0%)	28 (0.7%)
CKD	1,026 (2.0%)	46 (0.2%)	317 (1.6%)	357 (5.1%)	306 (7.2%)
Major depressive disorder	410 (0.8%)	85 (0.4%)	194 (1.0%)	82 (1.2%)	49 (1.1%)
Liver cirrhosis	197 (0.4%)	21 (0.1%)	95 (0.5%)	49 (0.7%)	32 (0.7%)
Number of NCDs	–	–	–	–	–
0	34,684 (67.6%)	19,197 (94.0%)	12,376 (63.2%)	2,235 (31.7%)	876 (20.5%)
1	9,156 (17.8%)	993 (4.9%)	4,500 (23.0%)	2,286 (32.4%)	1,377 (32.2%)
2	5,327 (10.4%)	198 (1.0%)	2,108 (10.8%)	1,748 (24.8%)	1,273 (29.8%)
≥ 3	2,177 (4.2%)	39 (0.2%)	603 (3.1%)	789 (11.2%)	746 (17.5%)

CKD = chronic kidney disease; COPD = chronic obstruction pulmonary disease; NCD = noncommunicable chronic disease; SES = socioeconomic status; 1 U.S. dollar = 30 New Taiwan Dollars.

**Table 2:** Adverse outcomes and health expenditure of patients with dengue in Taiwan, 2014–2015

	Total	Age (years)			
	(*N* = 51,344)	18–45 (*N* = 20,427, 39.8%)	45–65 (*N* = 19,587, 38.1%)	65–75 (*N* = 7,058, 13.7%)	≥75 (*N* = 4,272, 8.3%)
Health outcome (N, %)					
Hospitalization	15,847 (30.9%)	4,314 (21.1%)	5,471 (27.9%)	3,233 (45.8%)	2,829 (66.2%)
Mortality	373 (0.7%)	8 (0.0%)	53 (0.3%)	114 (1.6%)	198 (4.6%)
ICU use	894 (1.7%)	62 (0.3%)	163 (0.8%)	304 (4.3%)	365 (8.5%)
Resource utilization (Mean, STD)	–	–	–	–	–
Length of stay, days	6.11 (5.09)	5.14 (2.71)	5.60 (4.21)	7.03 (5.53)	8.79 (7.84)
Inpatient care expenditure, USD	782.65 (1,876.75)	484.45 (1,248.14)	624.39 (1,239.45)	1,059.75 (2,212.71)	1,438.71 (2,870.80)
Total medical care expenditure, USD	379.27 (1,358.62)	198.75 (964.52)	327.02 (976.81)	708.51 (1,798.23)	1,270.75 (2,668.23)

ICU = intensive care unit. Binary variables are presented in n (%) and continuous variables are presented in mean (STD). 1 U.S. dollar = 30 New Taiwan Dollars. Average inpatient expenditure was calculated as the average inpatient expenditure of hospitalized patients only.

**Figure 3.**
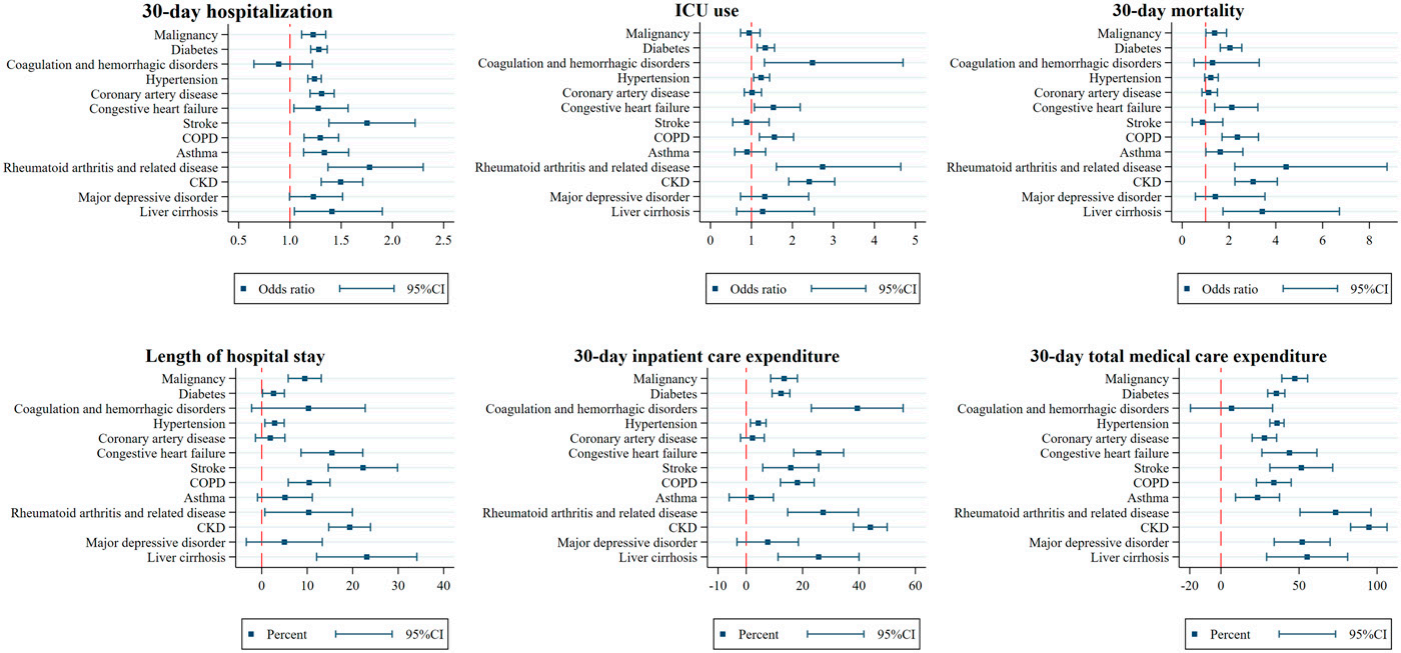
Factors associated with adverse outcomes and resource utilization for patients with dengue. OR = odds ratio. ICU = intensive care unit. COPD = chronic obstruction pulmonary disease; CKD = chronic kidney disease. All the regression controlled for sex, age, socioeconomic status, and the index year. For each noncommunicable disease, the reference group was the patients who did not have that disease. The unit in length of hospital stay was days, and in 30-day inpatient care expenditure and in total medical care expenditure were log-transformed and is presented in percent (%). This figure appears in color at www.ajtmh.org.

Figure 3 illustrates that after adjusting for patients’ age, sex, SES, and the index year, compared with patients without an underlying condition, patients who had RA and related disease (OR: 1.78; 95% CI: 1.37–2.30) had the highest risk of hospitalization within 30 days of dengue diagnosis, followed by stroke (OR: 1.75; 95% CI: 1.38–2.22), CKD (OR: 1.49; 95% CI: 1.30–1.71), liver cirrhosis (OR: 1.41; 95% CI: 1.04–1.90), asthma (OR: 1.34; 95% CI:1.13–1.57), coronary artery disease (OR: 1.31; 95% CI:1.20–2.43), COPD (OR: 1.30; 95% CI: 1.14–1.47), diabetes (OR: 1.28; 95% CI: 1.20–1.36), CHF (OR: 1.28; 95% CI: 1.04–1.57), hypertension (OR: 1.24; 95% CI: 1.17–1.31), and malignancy (OR: 1.23; 95% CI: 1.11–1.35). Among hospitalized patients, those with coexisting RA and related disease (OR: 2.73; 95% CI: 1.61–4.65) had the highest risk of ICU use, followed by coagulation and hemorrhagic disorders (OR: 2.49; 95% CI: 1.32–4.70), CKD (OR: 2.41; 95% CI: 1.91–3.03), COPD (OR: 1.56; 95% CI: 1.19–2.03), CHF (OR: 1.53; 95% CI: 1.07-2.19), diabetes (OR: 1.34; 95% CI: 1.14–1.56), and hypertension (OR: 1.23; 95% CI: 1.05–1.44). Importantly, a higher risk for all-cause mortality within 30 days of dengue diagnosis was found in patients with coexisting RA and related disease (OR: 4.44; 95% CI: 2.25–8.76), liver cirrhosis (OR: 3.42; 95% CI: 1.74–6.72), CKD (OR: 3.03; 95% CI: 2.25–4.07), COPD (OR: 2.36; 95% CI: 1.70–3.26), CHF (OR: 2.12; 95% CI: 1.39–3.23),diabetes (OR: 2.04; 95% CI: 1.63–2.55), asthma (OR: 1.62; 95% CI: 1.01–2.59), and malignancy (OR: 1.38; 95% CI: 1.01–1.89).

In terms of resource utilization, patients who had coexisting liver cirrhosis had a significant 23.1% increase in the average LOS, followed by stroke (22.2%), CKD (19.3%), CHF (15.4%), COPD (10.4%), RA and related disease (10.3%), malignancy (9.5%), hypertension (2.8%), and diabetes (2.6%). Coexisting CKD significantly increased the average inpatient expenditure by 44.0%, followed by coagulation and hemorrhagic disorders (39.4%), RA and related disease (27.2%), CHF (25.7%), liver cirrhosis (25.6%), COPD (18.1%), stroke (15.8%), malignancy (13.4%), diabetes (12.3%), and hypertension (4.3%). Patients with CKD had a significant 94.7% higher average total medical expenditures, followed by RA and related disease (73.4%), liver cirrhosis (55.2%), major depressive disorder (52.0%), stroke (51.4%), malignancy (47.2%), CHF (43.8%), hypertension (35.8%), diabetes (35.4%), COPD (33.8%), coronary artery disease (27.8%), and asthma (23.4%).

**Figure 4.**
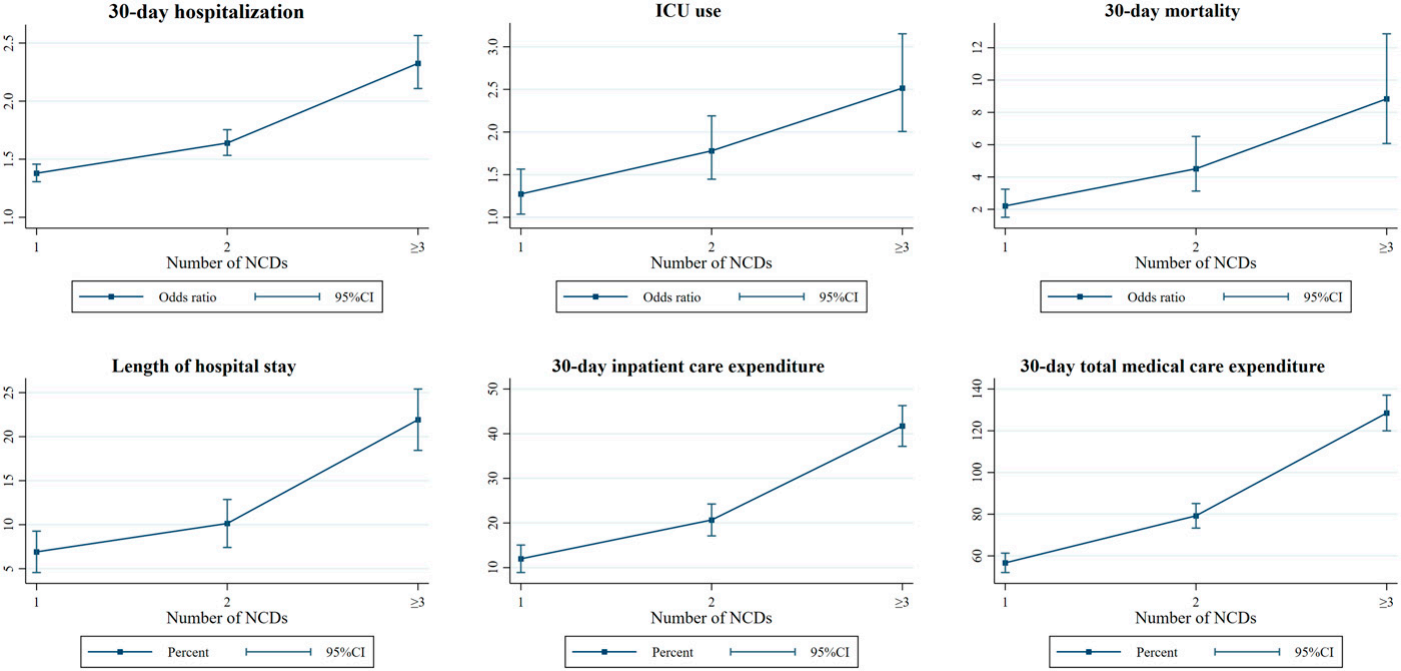
Number of noncommunicable diseases associated with adverse outcomes and resource utilization for patients with dengue. OR = odds ratio; ICU = intensive care unit. All the regression controlled for sex, age, socioeconomic status, and the index year. The reference group was the patients who did not have any of noncommunicable disease. The unit in length of hospital stay was days, and in 30-day inpatient care expenditure and in total medical care expenditure were log-transformed and is presented in percent (%). This figure appears in color at www.ajtmh.org.

**Figure 5.**
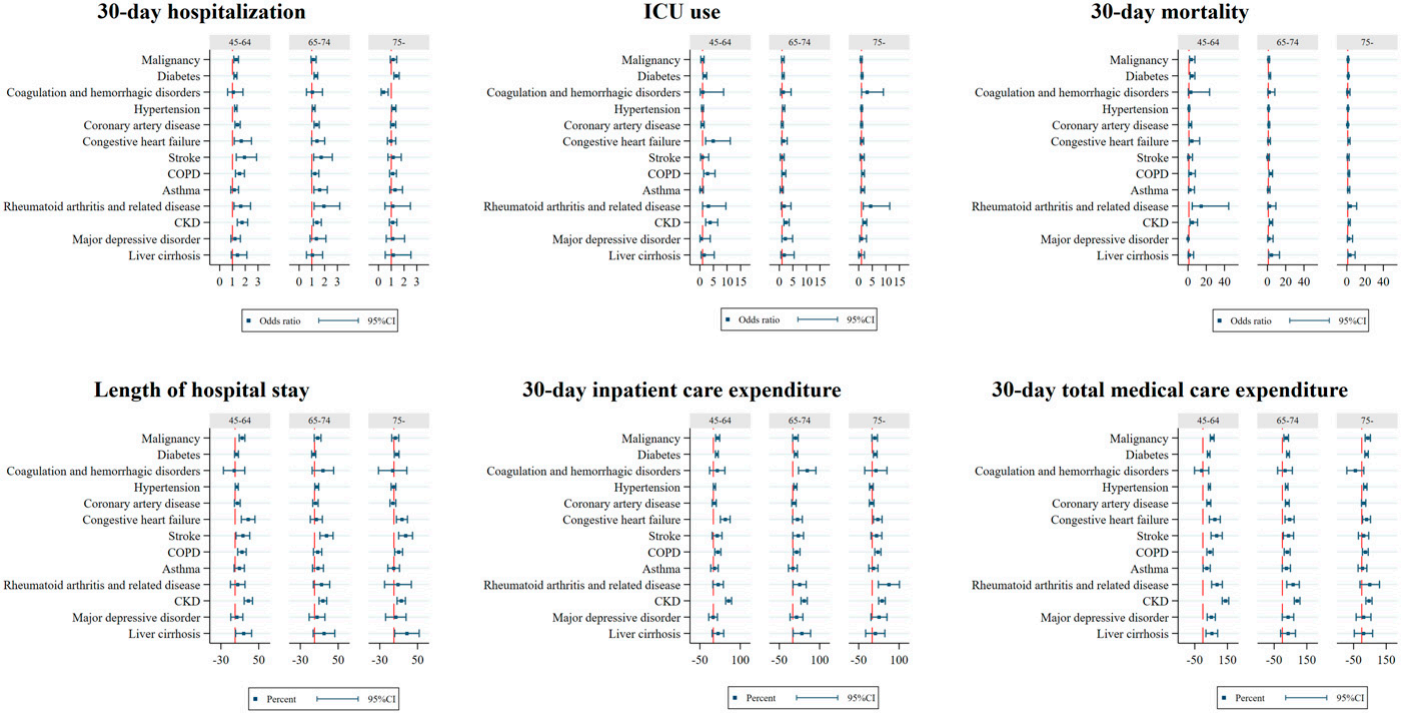
Factors associated with adverse outcomes and resource utilization for patients with dengue, stratified by age. OR = odds ratio; ICU = intensive care unit; COPD = chronic obstruction pulmonary disease; CKD = chronic kidney disease. All the regression controlled for sex, age, socioeconomic status, and the index year. For each noncommunicable disease, the reference group was the patients who did not have that disease. The unit in length of hospital stay was days, and in 30-day inpatient care expenditure and in total medical care expenditure were log-transformed and is presented in percent (%). This figure appears in color at www.ajtmh.org.

The results also indicated that after adjusting for other variables, dose-response patterns were observed among the number of coexisting NCDs, risk of adverse outcomes, and resource utilization (Figure 4) with higher risk for adverse outcomes and more resources used with increasing number of underlying conditions. Similar dose-response patterns were consistently observed across all groups of patients aged 45 years or above. Risks for individual outcomes by NCD and age strata are provided in Figure 5.

## DISCUSSION

This study confirmed that specific underlying chronic diseases exacerbate the course of acute dengue, resulting in a higher frequency of and prolonged duration of hospitalization, greater need for ICU care, and a higher risk of fatal outcome. These results will sound familiar in the context of the COVID-19 pandemic, which has highlighted the role of host factors, prominently, advanced age, obesity, and some of the same underlying diseases discovered in our analysis, as risk factors for severe outcomes and death. We initiated the study before the pandemic’s emergence, prompted by previously reported case series that highlighted complicated outcomes of dengue in patients with specific NCDs and by the long-known role of chronic diseases as risk factors for severe influenza. Dengue has emerged as yet another acute viral infection for which adverse outcomes appear to be associated with underlying NCDs. The growing prevalence of NCDs globally, including in tropical and subtropical regions where dengue is endemic, overlaying the ongoing secular trend for dengue to be acquired later in life, suggests that the medical burden of dengue increasingly will be comprised of cases complicated by underlying chronic diseases.

We first showed that the cause of complicated and severe dengue was less likely to be attributed to the plasma leakage and hemorrhagic syndrome associated with secondary dengue; although, we cannot rule out some patients being miscoded because physicians may not have adapted to a change in the DHF/DSS codes in 2015. This is unsurprising as dengue is not transmitted in an endemic pattern in Taiwan. The infection is introduced periodically by travelers and outbreaks occur irregularly and typically are limited and the last major outbreak before the 2014–2015 epidemic was in 2002–2003. The majority of adults that remained dengue naive making the secondary infection a less significant cause of severe disease.^21^

We showed that a range of antecedent chronic illnesses conferred a risk for dengue cases necessitating hospitalization, ICU care, or resulting in death; some of these conditions had been identified as risk factors in previous case series, but we also identified disorders of coagulation and hematological conditions and cirrhosis were more likely to be associated with adverse outcome. Interestingly, while eight of the examined conditions increased risk for hospitalization, illness of a severity necessitating ICU care occurred only in patients with underlying coagulation or hematologic disorders, rheumatic diseases, and CKD. At the same time, an increased risk for death in the 30 days after dengue diagnosis occurred in patients with underlying malignancy, CHF, COPD, asthma, rheumatic diseases, CKD, and cirrhosis; the last was not associated with an increased risk for hospitalization. We did not explore the hospital records of patients and can only speculate on the processes leading to an increased risk for complications and death among these patients.

Thrombocytopenia and capillary fragility in dengue can result in mucosal bleeding to a point that platelet transfusions previously were advocated. The LOS of patients with underlying coagulative disorders and higher rate of ICU admission may reflect bleeding complications associated with their care. Sickle cell anemia (SS) and sickle hemoglobin-C disease (SC), and glucose-6-phosphate dehydrogenase (G6PD) deficiency have been reported to be associated with increased dengue severity; though SS and SC traits are uncommon in Taiwan, G6PD deficiency is prevalent. Frailty associated with advanced age and disability is known to contribute to an increased risk for death after acute illness and hospitalization with influenza; the increased 30-day postdengue mortality in dengue patients with various chronic conditions (e.g., CHF, COPD, cirrhosis), also could reflect a delayed impact of an acute illness with similar severity. Our observation describing a dose-response relationship between the number of NCDs and adverse dengue outcomes, is consistent with this connection to frailty.

There are a few unique features of the data used in this study. First, the NDDCC records PCR-confirmed dengue cases. The fine imposed on providers in case of nonreporting ensures reporting rates as high as 96%.^22^ Second, the linkage with Taiwan’s NHIRD, which covers more than 99.5% of the population,^18^ enables us to identify chronic comorbidities that preexisted before the dengue episode. Third, the study period coincided with a large dengue epidemic,^23^ when clinical suspicion of the diagnosis in all age groups was high and laboratory confirmation of dengue likely would have been sought in most cases. The size of the outbreak 51,433 officially reported dengue cases, provided the largest sample size for analysis among similar studies. The relatively short study period, from 2014 to 2015, plausibly provided consistency in providers’ admission criteria, quality of care, and the patients’ health-seeking behaviors, that otherwise might have been difficult to control, jeopardizing the internal validity of the variables of interests, for example, mortality, hospitalization, and ICU stay. For example, it is likely that in a situation of endemic dengue transmission, clinicians might not consider the diagnosis in patients without signs and symptoms associated with “classical” dengue, particularly in adults. Lastly, the linked data sources allowed us to control for SES of each studied individual, as the role of SES as a risk factor for acquiring dengue has been controversial.^24^^,^^25^

Few studies have investigated the association between RA and dengue outcomes. However, among the NCDs we investigated, RA and related conditions were associated with the highest ORs for hospitalization, ICU stays, and dengue-related mortality, corroborating previous findings associating RA with increased risk for dengue hospitalization and death.^26^ (Note that the definition of “related conditions” does not include other connective tissue diseases but refers to other manifestations of RA). Our study did not examine concurrent medications used to treat underlying NCDs and the potential contribution of immunomodulators in these outcomes; this should be clarified in future studies.

The COVID-19 outbreak has highlighted the contribution of underlying obesity and diabetes as risk factors for severe illness even as its contribution to increased severity of influenza has been well recognized. However, strong epidemiological evidence that suggests or quantifies such association is scarce.^27^ It is biologically plausible that the pro-inflammatory baseline status and endothelial cell dysfunction of diabetes patients could predispose them to a more severe course of dengue, which itself is associated with increased vascular permeability and cytokine activation.^28^ Fluid management of dengue may be more complicated in diabetic patients. Our study results corroborate and expand on previous conclusions that diabetes independently predicted hospitalization (OR: 1.17; 95%CI: 1.09–1.25), ICU use (OR: 1.29; 95%CI: 1.05–1.59), and dengue-related mortality (OR: 1.87; 95% CI: 1.43–2.45).^29^ Several plausible biological mechanisms may help explain how underlying CKD may lead to complications that result in severe and fatal outcomes. The infection itself could be more difficult to control as patients with CKD may be relatively immunocompromised, while a uremic milieu contributes to further endothelial cell and platelet dysfunction. Pathophysiological consequences of dengue infection, especially plasma leakage leading to reduced intravascular volume could aggravate already compromised renal function and would challenge fluid and electrolyte management, not only in patients with CKD but also in those with CHF.^30^

The analysis, contrary to expectations, did not find an association between chronic hepatitis and the adverse outcomes that we explored. Elevations in hepatic transaminases and acute hepatitis frequently occur during acute dengue, to the point that acute liver failure in dengue has been managed with liver transplantation. We expected to but, did not see, the phenomenon of acute on chronic hepatitis expressed in increased risks for hospitalizations and LOS, if not mortality. However, more advanced liver disease, cirrhosis, was associated with an increased risk for death in this analysis.

Among the limitations of the analysis, it is possible that increased risks for death within 30 days of dengue diagnosis in patients with cirrhosis, malignancy and possibly, other conditions, was due to the use of a reference group of healthier patients without NCDs and was unrelated to acquiring dengue. Due to inherited limitations of the administrative data and the NHI claims data, we could not control for all confounders. Elevated BMI, which is unavailable in the NHIRD and which has been associated with more severe dengue may have confounded associations with diabetes and other conditions. The dataset also does not distinguish primary from secondary dengue infection or the infection serotype, though the outbreak was dominated by two imported viral strains of dengue 1 and dengue 2. Our estimations of medical expenditures did not include medications paid out-of-pocket by patients. Although ascertainment of confirmed dengue cases in the NICDD is high, underreporting, especially of mild cases cannot be ruled out. Lastly, because of heterogeneity in medical practices and healthcare systems across countries, the findings may not be generalizable to other parts of the world.

## Supplemental Material


Supplemental materials
