# Supplementary material for: A Population-Based Cohort Study on Chronic Comorbidity Risk Factors for Adverse Dengue Outcomes
Source: Am J Trop Med Hyg. 2021 Sep 27;105(6):1544–51. doi: 10.4269/ajtmh.21-0716 (PMC8641312; doi:10.4269/ajtmh.21-0716)
Supplement: Supplementary file 1 [file tpmd210716.SD1.pdf]

Supplemental Table 1. Univariate analysis for each NCD

	Health outcome			Resource utilization		
	Hospitalization	Mortality	ICU use	Length of stay	Inpatient care expenditure	Total medical care expenditure
	aOR (95% CI)	aOR (95% CI)	aOR (95% CI)	Percent (95% CI)	Percent (95% CI)	Percent (95% CI)
**Comorbidities**						
Malignancy	1.29 (1.18 - 1.42)	1.78 (1.32 - 2.40)	1.29 (1.18 - 1.42)	11.75 (8.11 - 15.39)	18.06 (13.27 - 22.85)	29.68 (14.67 - 44.69)
Diabetes	1.43 (1.35 - 1.52)	2.46 (1.99 - 3.04)	1.43 (1.35 - 1.52)	5.79 (3.45 - 8.13)	18.08 (15.01 - 21.15)	39.12 (29.46 - 48.78)
Coagulation and hemorrhagic disorders	1.02 (0.75 - 1.39)	1.93 (0.77 - 4.80)	1.02 (0.75 - 1.39)	17.76 (5.25 - 30.28)	51.07 (34.59 - 67.56)	-7.02 (-55.10 - 41.06)
Hypertension	1.38 (1.32 - 1.46)	1.62 (1.30 - 2.03)	1.38 (1.32 - 1.46)	5.47 (3.43 - 7.52)	10.35 (7.66 - 13.05)	35.96 (28.05 - 43.87)
Coronary artery disease	1.49 (1.37 - 1.63)	1.45 (1.10 - 1.91)	1.49 (1.37 - 1.63)	5.39 (2.21 - 8.58)	8.64 (4.44 - 12.85)	53.93 (39.90 - 67.96)
Congestive Heart Failure	1.65 (1.35 - 2.02)	3.29 (2.22 - 4.87)	1.65 (1.35 - 2.02)	21.93 (15.22 - 28.65)	37.66 (28.81 - 46.51)	103.36 (71.57 - 135.16)
Stroke	2.15 (1.70 - 2.72)	1.30 (0.66 - 2.57)	2.15 (1.70 - 2.72)	26.70 (19.09 - 34.31)	25.67 (15.63 - 35.70)	90.25 (53.54 - 126.96)
COPD	1.44 (1.27 - 1.63)	2.63 (1.94 - 3.56)	1.44 (1.27 - 1.63)	13.53 (9.03 - 18.03)	22.05 (16.12 - 27.98)	53.04 (33.11 - 72.97)
Asthma	1.52 (1.30 - 1.78)	2.36 (1.52 - 3.67)	1.52 (1.30 - 1.78)	9.68 (3.76 - 15.60)	10.09 (2.28 - 17.89)	41.80 (16.53 - 67.07)
Rheumatoid arthritis and related disease	1.82 (1.40 - 2.35)	4.27 (2.19 - 8.32)	1.82 (1.40 - 2.35)	11.51 (1.82 - 21.20)	29.35 (16.58 - 42.13)	71.55 (29.85 - 113.25)
CKD/Dialysis	1.77 (1.55 - 2.02)	4.17 (3.15 - 5.52)	1.77 (1.55 - 2.02)	22.93 (18.38 - 27.47)	51.63 (45.67 - 57.58)	84.22 (63.11 - 105.32)
Major depressive disorder	1.30 (1.06 - 1.60)	1.28 (0.52 - 3.14)	1.30 (1.06 - 1.60)	6.33 (-2.09 - 14.74)	9.11 (-1.98 - 20.20)	35.24 (2.42 - 68.05)
Liver cirrhosis	1.64 (1.22 - 2.20)	4.91 (2.59 - 9.34)	1.64 (1.22 - 2.20)	30.08 (19.08 - 41.07)	40.71 (26.21 - 55.20)	-18.47 (-65.73 - 28.78)
**NCDs**						
0	1 (ref)	1 (ref)	1 (ref)	1 (ref)	1 (ref)	1 (ref)
1	1.37 (1.30 - 1.45)	2.23 (1.52 - 3.28)	1.37 (1.30 - 1.45)	4.72 (2.15 - 7.28)	-0.06 (-7.27 - 7.15)	36.80 (28.31 - 45.29)

	Health outcome			Resource utilization		
	Hospitalization	Mortality	ICU use	Length of stay	Inpatient care expenditure	Total medical care expenditure
	aOR (95% CI)	aOR (95% CI)	aOR (95% CI)	Percent (95% CI)	Percent (95% CI)	Percent (95% CI)
2	1.63 (1.52 - 1.74)	4.56 (3.16 - 6.59)	1.63 (1.52 - 1.74)	7.55 (4.58 - 10.51)	6.68 (-1.66 - 15.02)	55.86 (45.11 - 66.61)
≥3	2.30 (2.09 - 2.54)	9.01 (6.19 - 13.11)	2.30 (2.09 - 2.54)	14.99 (11.22 - 18.76)	6.25 (-4.33 - 16.84)	100.03 (84.40 - 115.66)

OR=odds ratio. ICU=Intensive care unit. COPD=Chronic obstruction pulmonary disease. CKD=Chronic kidney disease. All the regression controlled for sex, age, socioeconomic status, and the index year. For each non-communicable disease, the reference group was the patients who did not have that disease. The unit in length of hospital stay was days, and in 30-day inpatient care expenditure and in total medical care expenditure were log-transformed and should be explained in percent (%).
